# How does timing of flowering affect competition for pollinators, flower visitation and seed set in an early spring grassland plant?

**DOI:** 10.1038/s41598-019-51916-0

**Published:** 2019-10-30

**Authors:** Sandra Kehrberger, Andrea Holzschuh

**Affiliations:** Department of Animal Ecology and Tropical Biology, Biocenter, University of Würzburg, Am Hubland, 97074 Würzburg, Germany

**Keywords:** Ecology, Ecology

## Abstract

Knowledge on how the timing of flowering is related to plant fitness and species interactions is crucial to understand consequences of phenological shifts as they occur under climate change. Early flowering plants may face advantages of low competition for pollinators and disadvantages of low pollinator abundances and unfavourable weather conditions. However, it is unknown how this trade-off changes over the season and how the timing affects reproductive success. On eight grasslands we recorded intra-seasonal changes in pollinators, co-flowering plants, weather conditions, flower visitation rates, floral longevity and seed set of *Pulsatilla vulgaris*. Although bee abundances and the number of pollinator-suitable hours were low at the beginning of the season, early flowers of *P. vulgaris* received higher flower visitation rates and estimated total number of bee visits than later flowers, which was positively related to seed set. Flower visitation rates decreased over time and with increasing number of co-flowering plants, which competed with *P. vulgaris* for pollinators. Low interspecific competition for pollinators seems to be a major driver for early flowering dates. Thus, non-synchronous temporal shifts of co-flowering plants as they may occur under climate warming can be expected to strongly affect plant-pollinator interactions and the fitness of the involved plants.

## Introduction

The optimal timing of flowering is crucial for plant fitness^1^. The timing of flowering depends on abiotic factors like temperature and the availability of water, nutrients and light^2,3^. Besides, interspecific interactions with mutualists, like pollinators, and competitors, like co-flowering plants, have been suggested to affect the timing of flowering^2–4^. In many plants that depend on animal pollination seed set increases with increasing pollinator visitation rates^5,6^. However, plants that are poor competitors for pollinators may receive reduced pollinator visitation rates in the presence of competing co-flowering plant species, which are more attractive to pollinators^7^. Previous studies indicated that plant species can mitigate negative effects of low pollinator visitation by elongating their floral longevity, which increases the probability of pollinator visitation, but warm temperatures may hinder elongation^8,9^. If plants cannot mitigate for low pollinator visitation rates then competition for pollinators could drive poor competing plants to shift their flowering phenology to times with less competition^7^. So competing plant species can achieve coexistence by temporal niche separation^10^. However, the options for plant species to shift flowering to periods with less competing plants being present, like at the beginning of the flowering season, are limited by pollinator availability^11,12^, temperatures allowing flower survival^13^ and foraging activity of pollinators^14,15^. The net outcome of those different drivers on the selection of flowering phenology depends not only on their direction, but also on their strength^16^. So far, we know little about the joint effects of temperature, pollinator availability and competition by co-flowering plants on pollinator visitation and the resulting reproductive success of plant species. We would expect a directional selection towards earlier flowering dates if reproductive success would increase with earlier flowering, but a more stabilizing selection if first and last flowers have lowest reproductive success compared to peak flowers.

Plant species flowering at the beginning of the season constitute good model organisms to study how these drivers and their joint effects change over time, if their flowering period covers a sufficiently long period with a strong change in the gradient of the drivers. Early flowering plants may face the risk of low pollinator availability and low temperatures. However, they may also have the advantage of a flowering onset in the absence of co-flowering plants, and thereby in the absence of interspecific competition for pollinators for a certain time span of flowering. Previous studies focusing on plants flowering as the first plant species in the season in forests and subalpine grasslands showed that the first flowers had a reduced seed or fruit set compared to later flowers. This was suggested to be caused by low pollinator availability due to either low pollinator abundances or impaired foraging activity by low temperatures^13,17–21^.

This raises the question why plant species don’t start flowering later, especially grassland plants, which are not restricted by canopy closure, like plants in deciduous forests. One explanation could be that competition with later co-flowering plants for pollinators may reduce reproductive success. For plant species in the Mediterranean, which start flowering in winter, it has been shown that pollinator visitation was lower after the flowering onset of co-flowering plant species, which were suggested to withdraw pollinators^22^. We suggest that competition with co-flowering plants for pollinators is lowest at the beginning of the season, but increases over time, which in turn decreases reproductive success. However, the net outcome of the advantages of low interspecific competition for pollinators and the disadvantages of low pollinator abundances and low numbers of hours with temperatures suitable for pollinator foraging on flower visitation rates and reproductive success and their change over time has not yet been studied. With climate warming temporal co-flowering patterns may change due to species-specific phenological responses to warmer temperatures^23^. This could alter the strength and/or the direction of the drivers acting on flower phenology and reproductive success. We suggest that to predict future effects of climate warming on plant communities knowledge about the current drivers and their strength and direction on flower phenology is necessary.

In this study we focused on the red-list spring plant *Pulsatilla vulgaris*, which is the first plant species to flower on semi-natural calcareous grasslands in Germany. On eight sites, we studied how the timing of flowering affected pollinator availability, competition for pollinators, pollinator visitation rates, floral longevity, the number of hours with temperatures allowing pollinator activity, the estimated total number of bee visits, and seed set.

We tested the following hypothesis:

1. Flower visitation rates of *P. vulgaris* flowers increase with increasing bee abundance, but decrease with increasing competition for pollinators with co-flowering plant species.
2. Later dates of bud opening shorten floral longevity, but increase the flower-specific number of pollinator-suitable hours with temperatures allowing pollinator activity and the estimated total number of bee visits per flower of *P. vulgaris*.
3. *P. vulgaris* benefits from insect pollination and seed set increases with an increase in the estimated total number of bee visits per flower and is higher for early than for late dates of bud opening.

## Results

The flowering period of naturally occurring *Pulsatilla vulgaris* populations started in week 11 or 12 of the year, depending on site, showed peak flowering in week 13 or 14 and ended in week 15 to 19. In total we observed 929 bee individuals from 54 bee species and 11 genera during transect walks. *P. vulgaris* was visited by 80 bees comprising 12 species and four genera (excluding *Halictus* and *Lasioglossum*, which are no pollinators of *P. vulgaris*, and unidentified bees). The managed honeybee *Apis mellifera* was most common with 52.50 % of the bee visits on *P. vulgaris*, followed by the genera *Osmia* (18.75 %), *Bombus* (17.50 %) and *Andrena* (11.25 %).

**Figure 1:**
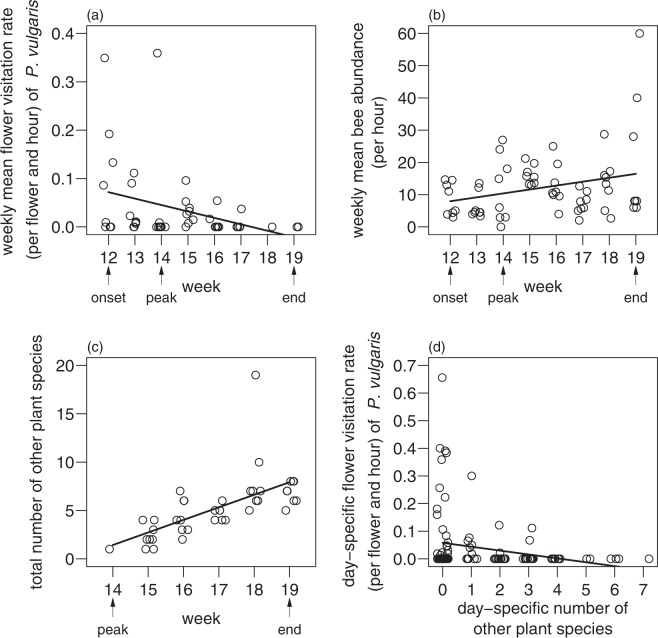
Relationship between week of the year and (**a**) weekly mean flower visitation rate (per flower and hour) of *P. vulgaris* flowers, (**b**) weekly mean bee abundance (per hour) on the transect and (**c**) total number of other flowering plant species on the transect. (**d**) Relationship between day-specific number of other flowering plant species and day-specific flower visitation rate (per flower and hour) of *P. vulgaris*. Results are from linear mixed-effects models. Solid lines show significant relationships (*p* < 0.05). Arrows show flowering onset (onset), peak flowering (peak) and flowering end (end) of *P. vulgaris*. A horizontal jitter was added to separate overlying data points.

In week 12 of the year, at the beginning of the flowering period of *P. vulgaris*, weekly mean flower visitation rate on *P. vulgaris* flowers was highest and decreased over time (linear mixed-effects model (lme): *F*_1,38_ = 5.3, *p* = 0.027, Fig. 1a). Contrary weekly mean bee abundance on the transect was lowest in week 12 and increased over time (lme: *F*_1,55_ = 6.0, *p* = 0.018, Fig. 1b). In week 14 other plant species than *P. vulgaris* started flowering. Besides *P. vulgaris* we recorded 20 flowering plant species during *P. vulgaris* flowering and 40 flowering plant species during the sampling period. The most common flowering plant species during *P. vulgaris* flowering were - besides *P. vulgaris* - *Potentilla neumanniana*, which occurred on all grasslands, followed by *Taraxacum officinale*, which occurred on seven grasslands, and *Euphorbia cyparissias* and *Viola sp*., which occurred on six grasslands. The total number of other flowering plant species increased over time (lme: *F*_1,32_ = 29.9, *p* < 0.001, Fig. 1c). With increasing day-specific number of other flowering plant species day-specific flower visitation rate on *P. vulgaris* flowers declined (lme: *F*_1,106_ = 6.9, *p* = 0.010, Fig. 1d).

**Figure 2:**
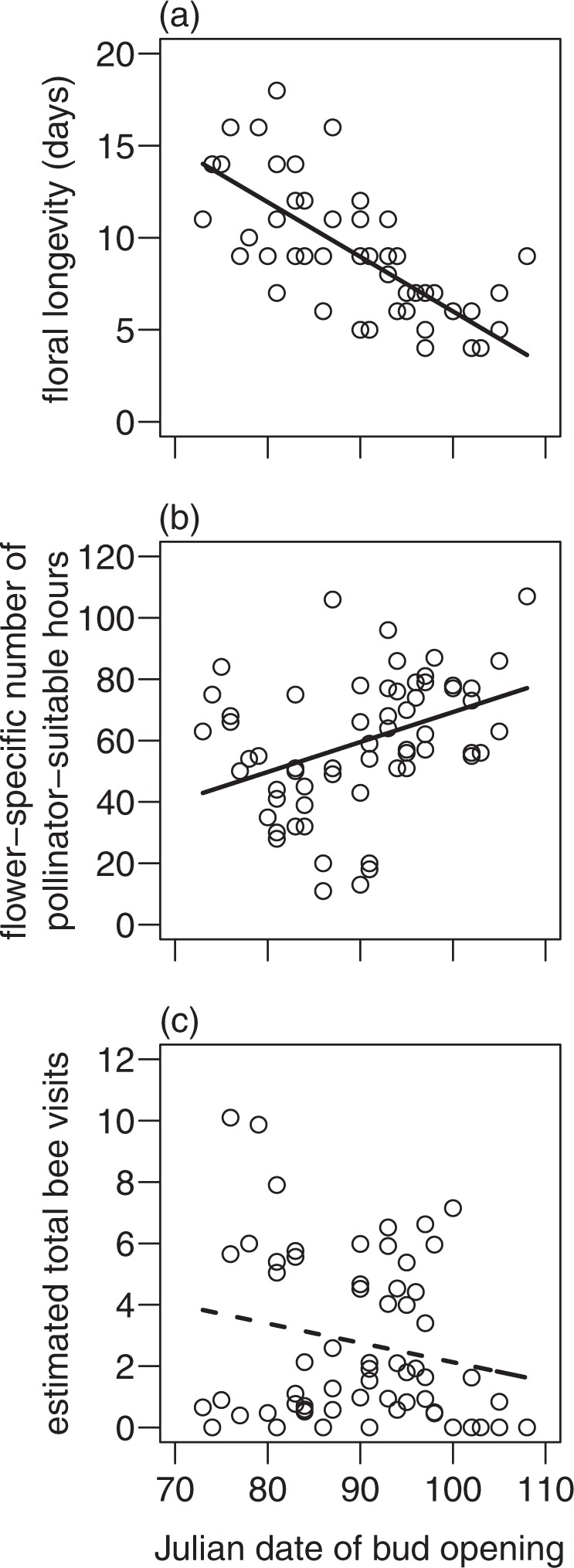
Relationship between Julian date of bud opening and (**a**) floral longevity (days), (**b**) flower-specific number of pollinator-suitable hours and (**c**) estimated total number of bee visits per flower of *P. vulgaris*. Solid lines show significant relationships (*p* < 0.05), dashed lines marginal significant relationships (*p* < 0.1).

Floral longevity of *P. vulgaris* shortened with later bud opening (lme: *F*_1,55_ = 78.5, *p < *0.001, Fig. 2a), as well as with increasing flower-specific mean temperature (lme: *F*_1,55_ = 22.4, *p < *0.001). Despite an increasing number of flower-specific pollinator-suitable hours with later bud opening (lme: *F*_1,55_ = 11.1, *p* = 0.002, Fig. 2b), the estimated total number of bee visits per flower marginally decreased with later bud opening (lme: *F*_1,55_ = 3.6, *p* = 0.065, Fig. 2c).

**Figure 3:**
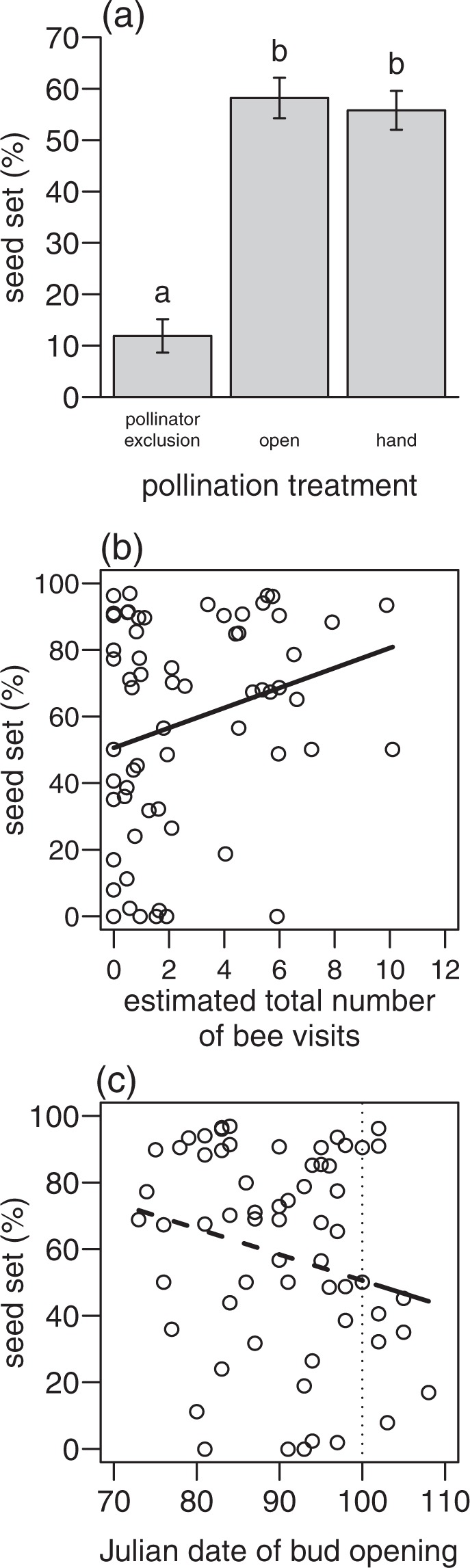
Relationship between (**a**) pollination treatment (pollinator exclusion (wind and self-pollination), open (wind, self- and insect pollination), hand (wind, self-, insect and hand pollination)) and mean seed set (±SE) of *P. vulgaris*. Relationship between (**b**) estimated total number of bee visits per *P. vulgaris* flower and (**c**) Julian date of bud opening and seed set of *P. vulgaris*. Different letters indicate significant differences (*p* < 0.05). The solid line shows a significant relationship (*p* < 0.05), the dashed line a marginal significant relationship (*p* < 0.1). The dashed, vertical line in (**c**) demonstrates the mean Julian date of flowering onset of other plant species.

The pollination experiment showed that pollinator exclusion (wind and self-pollination only) resulted in less than 20 % of the seed set produced by open flowers (wind, self- and insect pollination), whereas there was no difference between open and hand pollinated flowers in seed set (lme: *F*_2,164_ = 43.2, *p* < 0.001; post-hoc test: netted vs. open or hand: *p* < 0.001, open vs. hand: *p* = 0.643, Fig. 3a). Pollen limitation decreased with increasing seed set of open pollinated flowers (*F*_1,31_ = 10.4, *p* = 0.003, after removing one outlier: *F*_1,30_ = 31.2, *p* = < 0.001, Supplementary Fig. S1). Seed set increased with increasing estimated total number of bee visits for open pollinated flowers (lme: *F*_1,55_ = 4.1, *p* = 0.047, Fig. 3b).

A model testing the effects of Julian date of bud opening, pollination treatment (hand vs. open) and their interaction on seed set of *P. vulgaris* showed no significant effects (lme: Julian date of bud opening: *F*_1,118_ = 2.5, *p* = 0.113; treatment: *F*_1,118_ = 0.2, *p* = 0.664; Julian date of bud opening * treatment: *F*_1,118_ = 0.6, *p* = 0.426). However, when analysing seed set of hand and open pollinated flowers in separate models, seed set of open pollinated flowers marginally decreased with a later bud opening (lme: *F*_1,55_ = 2.8, *p* = 0.098, Fig. 3c), while seed set of hand pollinated flowers did not change with date of bud opening (lme: *F*_1,56_ = 0.6, *p* = 0.460).

## Discussion

Our study showed, that although bee abundance increased over time, flower visitation rates on *Pulsatilla vulgaris* flowers declined as the flowering season progressed. Furthermore, visitation rates on *P. vulgaris* flowers declined with increasing number of co-flowering plant species. The high attractiveness of *P. vulgaris* at the beginning of the flowering period was probably due to the absence of co-flowering plants and therefore of alternative food resources. The number of pollinator visits a flowering plant receives depends not only on the abundance of pollinators, but also on the attractiveness of the plant itself and of its co-flowering plants^24–26^. Our results show that the negative effect of the growing number of co-flowering plants and therefore the increase in interspecific competition for pollinators of *P. vulgaris* with its co-flowering plants could not be compensated by the increase in bee abundance, which led to an overall reduction of the flower visitation rates on *P. vulgaris* flowers over time.

Although the number of hours, which were suitable for pollinators to forage and therefore to visit a flower, increased over the season, the estimated total number of bee visits a *P. vulgaris* flower could receive during its flowering period did not increase, but marginally decreased. This decrease was caused by the decrease of flower visitation rates as well as the decrease of floral longevity over the season. The estimated value of the total number of bee visits for a *P. vulgaris* flower involves the flower-specific number of pollinator-suitable hours, the flower-specific mean flower visitation rate and the floral longevity and indicates how much visits would have been possible for a flower during its lifetime. However, the actual number of visits a flower received was probably lower, as the attractiveness of a flower to pollinators could have decreased over time or after successful pollination, due to a decline in floral scent or nectar production or due to withering^27^. Nevertheless, our results show that *P. vulgaris* could not enhance the floral longevity of late flowers when visitation rates were low. This is in contrast to other studies which showed that flowers can mitigate negative effects of low pollinator visitation rates by elongating their longevity^28,29^. We suggest that the shortage of the floral longevity of later *P. vulgaris* flowers seems to be imposed by the warming temperatures over the course of flowering, which probably enhance physiological processes like flower respiration and transpiration leading to a faster flower senescence^9^.

The decrease of the estimated total number of bee visits for a *P. vulgaris* flower over the season suggests that a higher number of pollinator-suitable hours and higher bee abundances later in the season could not compensate for the disadvantages arising during the season, namely the increasing competition for pollinators and the decrease of floral longevity. As a consequence of this, seed set of *P. vulgaris* was highest for the first flowers and marginally decreased over time. Contrary to our study, the seed set of herbal plants in deciduous forests and subalpine meadows was lowest in the first flowers but increased over time^13,17–21^. In our study, negative effects on flower visitation were overcompensated by the positive effect of low competition for pollinators at the beginning of the season. Our data strongly suggest that there is a causal relationship between the number of co-flowering plant species, pollinator visitation and seed set of *P. vulgaris*, however, we cannot exclude that other reasons than causal links might have resulted in the observed relationships. In general, flowering during periods of suboptimum flower visitation can be a bet-hedging strategy, where plants spread their flowering onset over time to buffer negative consequences of low visitation rates due to absent pollinator activity for the first flowers or due to pollinators drawn away by competing co-flowering plants for the last flowers. Our study shows for the first time that in cool temperate regions flowering as the first plant species of the season does not have to be negative for the reproductive success of early flowers, instead the last flowers were negatively affected. However, late flowers could act as insurance against rare, extreme cold weather events at the beginning of the flowering period, because the life time fitness of individuals is influenced by the reproductive success within multiple flowering seasons.

Seed set of open pollinated plants was more than five times as high as of plants where pollinators had been excluded. This confirms previous results that *P. vulgaris* strongly depends on insect pollination^30,31^. Self-fertilization in *P. vulgaris* is limited by the protogynous flowering schedule^32^. Pollen limitation was low if seed set of open pollinated flowers was high and vice versa suggesting that low seed set of open flowers was caused by high pollen limitation at that time. The seed set of *P. vulgaris* was positively correlated to the estimated total bee visits per flower. A previous study on *P. vulgaris*, which focused on 20 *P. vulgaris* flowers, indicated that 20 pollinator visits to a flower ensure a seed set of 90 %^31^. Our data suggest a threshold value of three total bee visits to ensure a seed set of more than 50 %. If a flower received less than three total bee visits during its flowering period, there was a high variance in the probability of successful and sufficient pollination. This high variance could be attributed to the circumstance that the amount of produced seeds is not only defined by the number of pollinator visits a flower receives, but also by the effectiveness^33^ and the functional differences of the flower visiting pollinators^34^.

We conclude that for plant species flowering at the beginning of the season in grasslands the limiting factor for reproduction seems to be low pollinator visitation imposed by interspecific competition for pollinators by co-flowering plants and not the low abundance of pollinators nor the limited time span for pollinators to forage due to unfavourable weather conditions. Climate warming, which advances the flowering onset of many plant species^35^, could negatively affect the reproductive success of *P. vulgaris* if co-flowering plants advance their flowering onset more strongly than *P. vulgaris*^23^. Our results suggest that future studies focussing on the effects of climate change on the reproductive success of plant species in a community, should not only consider non-parallel phenological shifts of plants and their pollinators^36^, but also changes in interspecific competition for pollinators. Another threat to the reproductive success of *P. vulgaris* could be the ongoing decline of pollinators^37,38^ and non-synchronous temporal shifts of flowering onset and pollinator emergence^39^.

## Materials and Methods

### Study sites

The study was conducted on eight calcareous grasslands around the city of Würzburg, Germany (49°47′ 28″N, 9°57′12″E). Grasslands had a minimum size of one hectar and were located in an area of about 116 km^2^ with a distance of 2.5 to 28.6 km between them. Calcareous grasslands comprise high biodiversity and rare species but are threatened by land use change as maintenance of grasslands depends on regular management^40^. Seven of the eight studied calcareous grasslands were managed by extensive sheep grazing, one was not managed. The population size of *Pulsatilla vulgaris* ranged between 15 and 600 individuals, depending on the site.

From 6^th^ February to 30^th^ May 2015 we hourly recorded air temperature with two temperature loggers per site (iButton temperature logger DS1922L, Maxim Integrated, USA; resolution: 0.0625 °C; Supplementary Note S2).

#### *Pulsatilla vulgaris*

The common pasque flower (*Pulsatilla vulgaris*; Ranunculaceae) is a perennial herb, which grows on calcareous grasslands^30^. On the studied sites it was the first herbal plant species that started flowering. *P. vulgaris* is listed as a threatened plant species in the red list of threatened plant species of Germany^41^. It reproduces sexually as well as vegetatively^30^. Flowering occurs between March and April^42^. During the flowering season *P. vulgaris* mostly produces one to three flowers per plant, which are hermaphrodite and protogynous^30^. Each flower is characterized by six purple-violet petals and numerous carpels and stamens, whereby the outer stamens are sterile and secreting nectar^43^. The main flower visitors of *P. vulgaris* are bees^31^. The produced seeds have a long feathery style and are dispersed by wind^30^.

### Data recording

To detect flowering onset of *P. vulgaris* populations we walked across each site between 6^th^ February and 4^th^ March 2015 every fourth to tenth day and after 4^th^ March 2015 every second to third day. *P. vulgaris* populations started flowering between 13^th^ and 18^th^ March depending on site and the last population ended flowering on 5^th^ May 2015. Bee and plant surveys were conducted between 17^th^ March and 5^th^ May 2015. During the sampling period, the phenology of *P. vulgaris*, of other than *P. vulgaris* flowering plant species and of bees (Apiformes) was recorded every second to third day on each site. Bees and the phenologies of other plant species than *P. vulgaris* were not recorded on days with daylong rain, but phenology of *P. vulgaris* was recorded. We conducted 21 bee surveys on five sites, 19 on two sites and 18 on one site. Due to different flowering durations of the *P. vulgaris* populations, *P. vulgaris* phenology surveys ranged from 13 to 24 surveys per site.

For each bee and plant survey, a variable transect of 100 m^2^ ^44^ containing the highest abundance of *P. vulgaris* flowers was chosen. We conducted bee and plant surveys until the flowering period of *P. vulgaris* populations had ended on all sites. If there were no more flowering *P. vulgaris* plants on the studied site, transects with the highest abundance of other flowering plants were chosen. We walked transects for 14 – 36 minutes (mean: 25 min.) per survey and recorded the number of bees and whether they were encountered on a *P. vulgaris* flower or at another location. Bees were captured if possible and individuals that could not be identified in the field were taken to the lab for further identification. To avoid multiple counts of single individuals, all in the field identified bees were released not until the end of the survey. Bees that could not be captured, were, if possible, classified to the genus level. We excluded bees that could not be assigned to a genus and the genera *Halictus* and *Lasioglossum* from the data set, because bees of these genera mostly never touched the carpels and may not have provided pollination services in our study. The lack of pollen transfer from *Halictus* and *Lasioglossum* bees to the carpels of *P. vulgaris* was confirmed by camera recordings of 56 *Halictus* and *Lasioglossum* bees (unpublished data). After each transect walk the number of other than *P. vulgaris* flowering plant species on the transect was recorded and the abundance of flowers or flower heads for each plant species including *P. vulgaris* estimated.

For *P. vulgaris* we calculated for every survey the day-specific flower visitation rate (per flower and hour), by dividing the number of bees on *P. vulgaris* flowers on the transect by the abundance of *P. vulgaris* flowers on the transect and the duration of the transect walk. The day-specific bee abundance (per hour) for every survey was calculated by dividing the number of all recorded bees on the transect by the duration of the transect walk. The weekly mean flower visitation rate on *P. vulgaris* flowers and the weekly mean bee abundance on the transect were calculated as the mean of the day-specific flower visitation rates and day-specific bee abundances, respectively. The day-specific number of other flowering plant species on the transect and the day-specific flower abundance of other plant species on the transect were highly correlated (Pearson rank correlation coefficient: *cor* = 0.83; *p* < 0.001), so we used only day-specific number of other flowering plant species on the transect for further analysis. The weekly total number of other flowering plant species was the cumulated number of other flowering plant species recorded on that site during one week.

### Pollination treatments and quantifying seed set

To identify the relative influence of wind, insect and optimal pollination on the seed set of *P. vulgaris* three pollination treatments were compared: (1) pollinator exclusion (wind and self-pollination only, mesh width of nets: 1 mm), (2) open flowers (wind, self- and insect pollination), (3) open hand-pollinated flowers (optimal pollination, wind, self-, insect and hand pollination). Every second or third day we randomly selected at least three *P. vulgaris* individuals per site, one for each treatment, and marked one up to two days old flower or bud per individual. The age of the flowers was determined by visual inspection. Young flowers are erect with a short stalk, have deep purple petals and are not yet fully opened^30,31^. We recorded the date of bud opening and the end of flowering for each marked flower or bud. For the pollinator exclusion treatment, we netted only closed flower buds. For the open and hand pollination treatments flowers were up to two days old. For the hand pollination treatment we randomly selected flowers of other *P. vulgaris* individuals with available pollen and removed the pollen with a brush. The pollen was deposited on the carpels of the hand pollinated flower by sweeping six times across the carpels with the pollen loaded brush. Pollen donator plants were marked and not used for further treatments. When no flower buds were available anymore, we ended treatments on that site. Treatments started at 16^th^ March 2015 and ended on four sites between 14^th^ and 17^th^ April 2015 and on three sites between 20^th^ and 24^th^ April 2015. On one site we could carry out treatments only on two days, 17^th^ and 22^nd^ March 2015 due to the small population size. In total we marked 81 flowers for the pollinator exclusion treatment, 80 flowers for the open pollination treatment and 78 flowers for the hand pollination treatment (Supplementary Table S3). Because of strong wind some nets opened during the study and, some flowers and seeds were broken off or partly eaten by animals, thus reducing the number of replicates to 45 flowers in the pollinator exclusion treatment, to 64 flowers in the open pollination treatment and 65 flowers in the hand pollination treatment. End of May we harvested the ripe seeds produced by the marked flowers and counted the number of fertilized and non-fertilized seeds, with non-fertilized seeds having shorter styli than fertilized^31^. Seed set (%) per flower was calculated by dividing the number of fertilized seeds by the sum of fertilized and non-fertilized seeds, which represents the number of ovules. Pollen limitation was calculated by dividing the seed set of hand pollinated flowers by the seed set of open pollinated flowers which had the same bud-opening date.

For each flower from the open pollination treatment we calculated the floral longevity, as the difference between the recorded date of flowering end and the recorded date of bud opening. The relationship between temperature during bee survey and day-specific flower visitation rate showed that bees were only observed at an air temperature warmer than 11.3 °C on *P. vulgaris* flowers. One exception was a single bumble bee queen, which was recorded at a temperature of 7.9 °C (Supplementary Fig. S4). This indicates that mainly temperatures warmer than 11.3 °C were suitable for bees to visit *P. vulgaris* flowers during our study. For every open pollinated flower we calculated the flower-specific number of pollinator-suitable hours, as the sum of hours equal or warmer than 11.3 °C between 8.00 am and 8.00 pm during flower life. We also calculated the flower-specific mean temperature as the mean of the hourly recorded temperatures during flower life. Furthermore we calculated the flower-specific mean flower visitation rate (per hour) as the mean of the day-specific flower visitation rates recorded during surveys with an air temperature equal or warmer than 11.3 °C during flower life. We estimated the total number of bee visits per flower for each open pollinated flower, by multiplying the flower-specific number of pollinator-suitable hours with the flower-specific mean flower visitation rate. For this estimation of the potential total number of bee visits a flower receives during flower life we assumed that the attractiveness of a flower stays constant until flowering end and that all hours equal or warmer than 11.3 °C were suitable for pollinators to forage.

### Statistical analyses

All statistical analyses were performed using the software R version 3.6.1^45^. For the linear-mixed effects models we used the nlme package^46^ and present p-values for Wald tests. To determine if the time of flowering affects the weekly mean bee visitation rate on *P. vulgaris* flowers (we used only weeks and sites with more than one *P. vulgaris* flower), the weekly mean bee abundance and the total number of other flowering plant species, we used linear mixed-effects models with week of the year as fixed factor and site as random factor. Out of 115 bee surveys, 58 took place when other plant species than *P. vulgaris* flowered on the transects. Using these surveys we tested the effect of the fixed factors day-specific number of other flowering plant species and Julian date on the response variable day-specific flower visitation rate on *P. vulgaris* flowers (only surveys with more than one *P. vulgaris* flower were used) with a linear mixed-effects model with site as random factor. Julian date was removed from the model because it had no additional explanatory power (p > 0.05) suggesting that it had no direct effects in addition to indirect effects via the number of other flowering plant species.

To test if floral longevity, flower-specific number of pollinator-suitable hours and the estimated total number of bee visits per flower of *P. vulgaris* are affected by bud opening we used linear mixed-effects models with site as random factor and Julian date of bud opening as fixed factor. As Julian date and mean temperature were positively correlated, we used a separate linear-mixed effects model with site as random factor, to analyse the effect of flower-specific mean temperature on the response variable floral longevity of *P. vulgaris*. When using both Julian date and flower-specific mean temperature as fixed factors, the latter had no additional explanatory power (p > 0.05). Furthermore, we analysed the effect of the fixed factor flower-specific mean temperature on the response variable floral longevity of *P. vulgaris* with a linear-mixed effects model with site as random factor.

We analysed if *P. vulgaris* depends on animal pollination with a linear mixed-effects model by comparing the effects of the fixed factor pollination treatment (pollinator exclusion vs. open vs. hand pollination) on the response variable seed set with site as random factor. The effect of pollination treatment was examined with the contrasts between the mean values of the three factor categories. To estimate the differences the glht function in the R-package multcomp^47^ was used. P-values of multiple comparisons were corrected by the Holm correction. To assess whether the seed set of open pollinated flowers was related to pollen limitation, we used linear mixed-effects models with seed set as fixed factor, pollen limitation as response variable and site as random factor. The effect of the fixed factor estimated total number of bee visits per flower on the response variable seed set of *P. vulgaris* was analysed with a linear mixed-effects model with site as random factor. When using both estimated total number of bee visits per flower and Julian date of bud opening as fixed factors in the same model, Julian date of bud opening had no additional explanatory power (p > 0.05) suggesting that date had an indirect effect by affecting the total number of bee visits. Furthermore, we tested the effect of the fixed factors Julian date of bud opening, pollination treatment (hand vs. open) and their interaction on the response variable seed set of *P. vulgaris* with a linear mixed-effects model with site as random factor. In a second step we tested the effect of the fixed factor Julian date of bud opening on the response variable seed set separately for open and hand pollinated *P. vulgaris* flowers. We visually inspected model residuals for violation of assumptions of normality and homoscedasticity.

## Supplementary information

Supplementary information

## Data Availability

All relevant data are deposited on Dryad at 10.5061/dryad.4b8gtht7t.
