# Supplementary material for: How does timing of flowering affect competition for pollinators, flower visitation and seed set in an early spring grassland plant?
Source: Sci Rep. 2019 Oct 30;9:15593. doi: 10.1038/s41598-019-51916-0 (PMC6821694; doi:10.1038/s41598-019-51916-0)
Supplement: Supplementary file 1 — Supplementary information [file 41598_2019_51916_MOESM1_ESM.pdf]

How does timing of flowering affect competition for pollinators, flower visitation and seed set in an early spring grassland plant?

Sandra Kehrberger, Andrea Holzschuh

Supplementary Figure S1

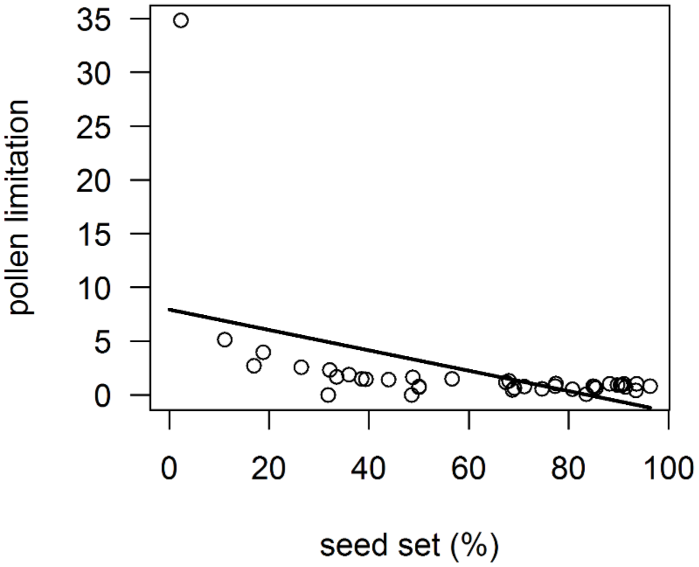

Fig. S1: Relationship between seed set (%) and pollen limitation. Pollen limitation decreased with increasing seed set of open pollinated flowers ($F_{1,31}$ = 10.4, p = 0.003, after removing one outlier: $F_{1,30}$ = 31.2, p = <0.001).

Supplementary Note S2

Methods of temperature recording: Loggers were fixed on two wooden posts each, 90 cm above ground, underneath a plastic tube, which was used for a separate experiment, and facing to south. The distance between the posts was five to 100 meters, depending on the site. For each site we calculated the hourly and the daily mean temperature as the mean of the two loggers. One logger failed on one site between 6th February and 21st March 2015 and one on another site between 6th February and 27th March 2015. Therefore the mean temperature for the two sites and time periods comprises only the temperature obtained from one logger.

Supplementary Table S3

Table S3: Mean, minimum (min.) and maximum (max.) number of marked flowers per site and marking days per site for the three pollination treatments pollinator exclusion, open pollination and hand pollination.

	marked flowers per site			marking days per site		
treatment	mean	min.	max.	mean	min.	max.
pollinator exclusion	10.1	2	14	9.5	2	13
open pollination	10.0	2	14	9.6	2	13
hand pollination	9.8	2	14	8.8	2	13

Supplementary Figure S4

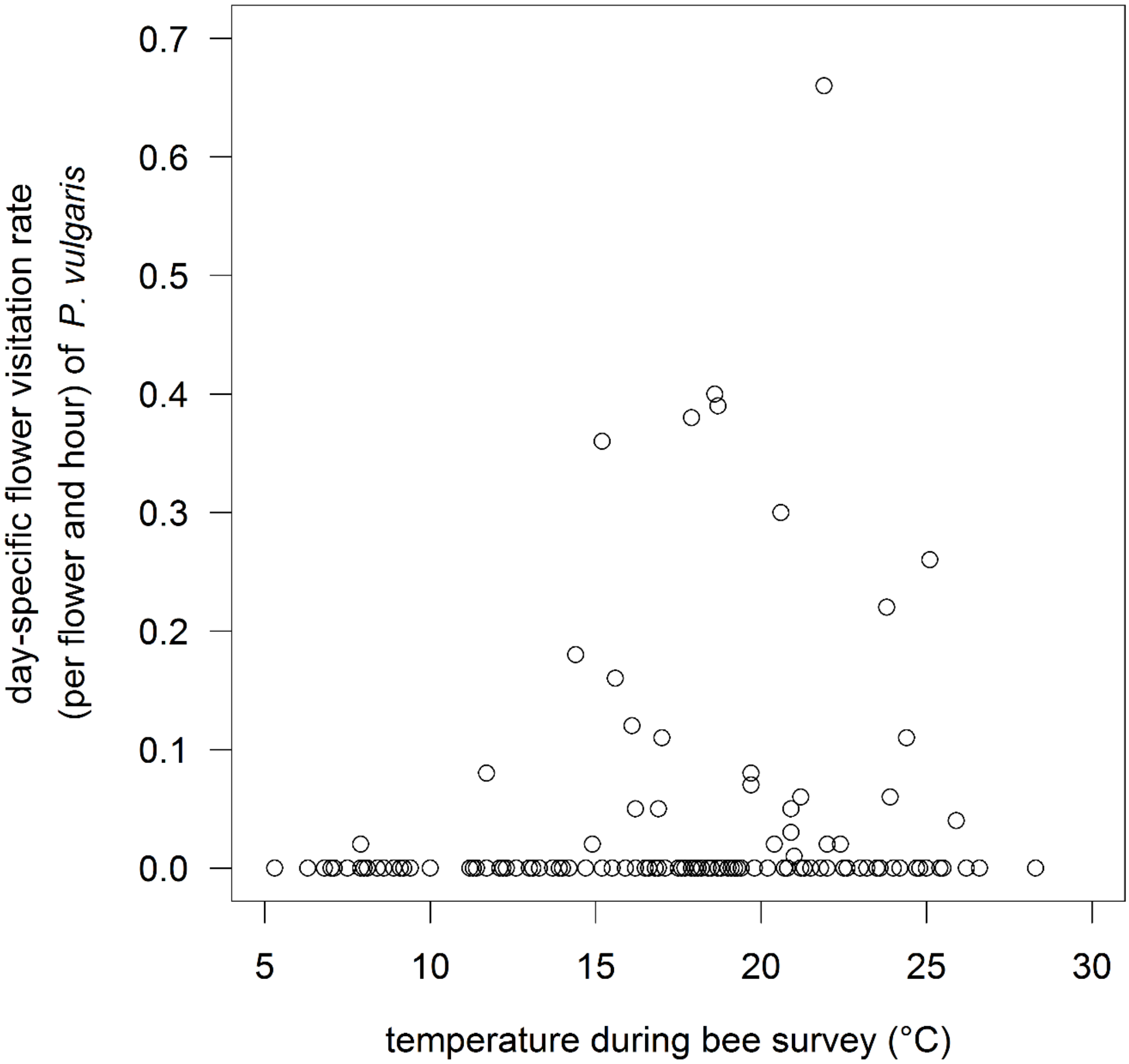

Fig. S4: Relationship between temperature (°C) during bee survey and day-specific flower visitation rate (per flower and hour) on *P. vulgaris*.
